# Uptake, distribution and radio-enhancement effects of gold nanoparticles in tumor microtissues†

**DOI:** 10.1039/d0na00256a

**Published:** 2020-06-02

**Authors:** Anna L. Neuer, Lukas R. H. Gerken, Kerda Keevend, Alexander Gogos, Inge K. Herrmann

**Affiliations:** Laboratory for Particles Biology Interactions, Swiss Federal Laboratories for Materials Science and Technology (Empa) Lerchenfeldstrasse 5 CH-9014 St. Gallen Switzerland inge.herrmann@empa.ch ingeh@ethz.ch +41 58 765 7153; Nanoparticle Systems Engineering Laboratory, Institute of Energy and Process Engineering, Department of Mechanical and Process Engineering, ETH Zurich Sonneggstrasse 3 CH-8092 Zurich Switzerland

## Abstract

Radiotherapy is an integral and highly effective part of cancer therapy, applicable in over 50% of patients affected by cancer. Due to the low specificity of the X-ray irradiation, the maximal radiation dose is greatly limited in order to avoid damage to surrounding healthy tissue. The limitations in applicable dose oftentimes result in the survival of a subpopulation of radio-resistant cells that then cause cancer reoccurence. Approaches based on tumor-targeted high atomic number inorganic nanoparticles have been proposed to locally increase the photoelectric absorption cross-section of tumors relative to healthy tissue. However, the complex interplay between the nanoparticle radio-enhancers and the tumor tissue has led to poor translation of *in vitro* findings to (pre)clinics. Here, we report the development of a tumor microtissue model along with analytical imaging for the quantitative assessment of nanoparticle-based radio-enhancement as a function of nanoparticle size, uptake and intratissural distribution. The advanced *in vitro* model exhibits key features of cancerous tissues, including diminished susceptibility to drugs and attenuated response to nanoparticle treatment compared to corresponding conventional 2D cell cultures. Whereas radio-enhancement effects between 2D and 3D cell cultures were comparable for 5 nm gold particles, the limited penetration of 50 nm gold nanoparticles into 3D microtissues led to a significantly reduced radio-enhancement effect in 3D compared to 2D. Taken together, tumor microtissues, which in stark contrast to 2D cell culture exhibit tissue-like features, may provide a valuable high-throughput intermediate pre-selection step in the preclinical translation of nanoparticle-based radio-enhancement therapy designs.

## Abstract 2

Differences in nanoparticle radio-enhancement efficiencies in 3D microtissues compared to conventional 2D cell cultures and contextualization with uptake and intratissural distribution data.

## Graphical abstract

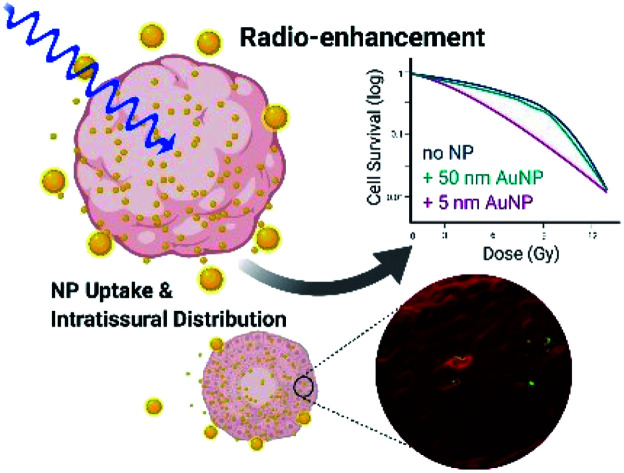

## Introduction

X-ray therapy is an integral part of cancer therapy plans. However, radiotherapy suffers from significant drawbacks, including limited efficiency, damage to nearby healthy tissue, radio-resistance effects, and increased risk for the development of secondary tumors.^1^ About 2–5% of secondary malignancies are attributable to previous radiation therapy.^2,3^ In order to increase the efficacy and specificity of radiotherapy, nanoparticle-based radio-enhancement has gained increasing interest.^4^ In nanoparticle-based radio-enhancement, secondary particles (such as photons and electrons) ejected from the nanoparticle surface upon irradiation initiate the generation of reactive oxygen species (ROS) in close proximity of the nanoparticles (NP).^5^ Depending on the sub-cellular localization, these reactive species can then cause damage to the DNA, proteins and other vital cellular building blocks.^6,7^ While elements with higher atomic number have higher X-ray absorption cross sections, Monte Carlo simulations have shown that the consideration of purely physical effects leads to underestimation of the eventual cellular damage initiated *via* X-ray-absorbing NP.^8^ Complex processes, including the reabsorption of secondary radiation may play a role in the eventual damage occurring. Experimental observations have revealed that the observed cell damage is the result of a combination of physical, chemical and biological effects. The therapeutic efficacy therefore depends on a multitude of parameters, including nanoparticle physico-chemical characteristics, sub-cellular localization, as well as cell-type-specific properties (*e.g.* DNA repair capacity).^4,5^ Different types of materials have already been investigated for their radio-enhancement properties, for example gold,^9^ platinum,^10^ hafnium dioxide,^11^ and magnetic microdiscs^12^ (see Kuncic *et al.*^4^ for a comprehensive review). Among these, gold nanoparticles (AuNP) are a natural choice as radio-enhancers due to their high atomic number, X-ray absorption cross section and biocompatibility, and are therefore the most widely researched nanoparticles to date.

Despite the overall promising results of nanoparticle-based radio-enhancement studies,^13^ the considerable variability of results (*e.g.* some studies showing significant radio-enhancement while others reporting radio-protection by the same nanomaterial),^14^ poses challenges to the rationalized therapy design and hampers translation into clinics. It is increasingly recognized that the lack of established standardized experimental approaches and thorough characterization of the experimental systems (*e.g.* NP uptake and distribution) are major obstacles for the translation of metal-based NP radio-enhancers.^15^ Additionally, most of the *in vitro* studies are performed on cell monolayers grown in culture plates in conditions significantly deviating from the native state of a tissue. Recent studies have shown that changing the cell microenvironment by culturing 3D microtissues (MT) instead of 2D monolayers has wide-ranging implications on the cellular phenotype and the response to drug exposure, mimicking the features of tumors much more accurately.^16–19^ Despite the initial promise of such advanced *in vitro* models in drug screening, the use of such models in the context of radiotherapy is poorly explored.^20–22^

In this study, we present nanoparticle-based radio-enhancement in a 3D tumor model featuring tissue-like properties. We investigate radio-enhancement effects of 5 and 50 nm-sized AuNP in this 3D tumor model in comparison to conventional 2D cell cultures. We present a label-free nano-analytical imaging route based on elemental analysis and density dependent color scanning electron microscopy (DDC-SEM), which enables in-depth characterization of the nanoparticle-containing MT and connection of the radio-enhancement results to NP uptake and intratissural distribution.

## Experimental section

### Cells and cell culture

HeLa, a human epithelioid cervix carcinoma cell line, was obtained from ATCC (Virginia, USA). Cells were cultured in Minimum Essential Medium (MEM#m2275, Sigma, Buchs, Switzerland) supplemented with 1% l-glutamine (Sigma-Aldrich, Buchs, Switzerland), 1% non-essential amino acids (NEAA, PAN Biotech, Aidenbach, Germany), 10% fetal calf serum (FCS, Sigma-Aldrich, Buchs, Switzerland), 1% Penicillin Streptomycin Neomycin (PSN, Sigma-Aldrich, Buchs, Switzerland) and 1 mM sodium pyruvate (Sigma-Aldrich, Buchs, Switzerland). Cells were sub-cultured every 2–3 days upon 80% confluence and cultured in a humidified incubator at 37 °C with 5% CO_2_ atmosphere (standard culture conditions).

NHDF, a non-cancerous human skin fibroblast cell line, was purchased from Sigma-Aldrich (Buchs, Switzerland). Cells were cultured in Dulbecco's Modifies Eagle Medium – high glucose (#RNBG3787, Sigma, Buchs, Switzerland) supplemented with 10% FCS, 1% PSN and 1% l-glutamine. Cells were sub-cultured once a week upon 80% confluence and medium was exchanged every 2–3 days. Cells were cultured under standard conditions.

### Nanoparticles

Citrate-stabilized AuNP (1 mg ml^−1^ in water) with sizes of 5 and 50 nm and a purity of 99.99% were purchased from nanoComposix (NanoComposix, San Diego, US). Two widely used citrate-capped gold nanoparticles with distinctly different tissue-penetration properties were chosen. For NP treatment NP, were directly pre-diluted in cell culture medium, added to the cells or microtissues and incubated for 24 h under standard culture conditions. Ultrapure water served as vehicle control.

### 3D cell culture

For 3D MT formation, the scaffold-free hanging drop technology from Insphero AG (Schlieren, Switzerland) was used. If not stated otherwise, 5000 HeLa cells were seeded per MT core, either using cells pre-treated with AuNP for 24 h or untreated cells. MT formation was performed in HeLa full growth medium containing 20% FCS. For HeLa/NHDF co-cultured MTs, NHDF cells were added on day 4 in NHDF full growth medium containing 20% FCS to the carcinoma MT core, resulting in a 1 : 1 mixture of HeLa : NHDF specified medium containing 20% FCS. MT were harvested on day 7 into a PBS pre-wetted GravityTRAP™ (Insphero AG Schlieren, Switzerland) 96-well plate for further growth and NP exposure. Medium was exchanged in both plates every 2–3 days with HeLa or HeLa–NHDF full growth medium containing 20% FCS. MT were cultured under standard culture conditions (37 °C, 5% CO_2_). The mean geometric diameter of the spherical MTs was calculated by the equation
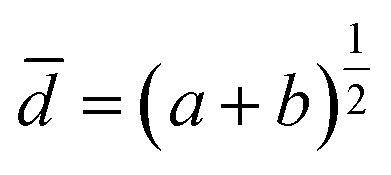
where *a* and *b* are the measured orthogonal diameters.

MT were further cultivated in GravityTRAP™ plates and exposed to AuNP for 24 h. If not stated otherwise, a particle dose of 20 μg per MT (4 ng per cell) was used.

### Histology

#### Paraffin embedding

MTs were fixed with 4% paraformaldehyde (PFA, Sigma-Aldrich, Buchs, Switzerland) and 3% glutaraldehyde (Sigma-Aldrich, Buchs, Switzerland) in PBS (phosphate buffered saline solution) for 2 h at room temperature (RT) and overnight at 4 °C. Prior to paraffin embedding MT were pooled, washed with 50% ethanol (EtOH) and pre-embedded in 3% agarose (Sigma-Aldrich, Buchs, Switzerland). Dehydration and paraffin embedding was performed with a Myr Spin Tissue Processor STP120 (Tarragona, Spain) using a total duration of 3 h for the dehydration and paraffin embedding.

#### Sectioning

A microtome (RM2235, Leica Biosystems, Wetzlar, Germany) was used for sectioning. Paraffin sections of 4 μm thickness were cut and transferred to X-tra™ adhesive slides (Leica Biosystems, Wetzlar, Germany).

#### Hematoxylin & Eosin (HE) staining

Deparaffinization and rehydration was performed by inverse xylene and decreasing EtOH concentration gradient steps. HE staining was performed by incubating the samples for 5 min in Hematoxylin and 30 s in Eosin solution. For preservation, the samples were dehydrated in steps (successively 70%, 80%, 90%, 96%, 100% EtOH, 2% isopropanol, 2× xylene, 2 min each) and mounted for storage. A ZEISS Primovert Microscope with an Axiocam 105 (Zeiss, Feldbach, Switzerland) color camera was used for image acquisition.

### Fluorescent antibody staining

MTs were fixed as described above. Triton X-100 was added at RT at a final concentration of 0.1% in PBS. Staining was performed before embedding. 5% BSA blocking in PBS was applied for 1 h at RT. A fibroblast specific TE7 primary antibody (AB) (TE7, Merk Millipore, #CBL271) was applied 1 : 100 in 1% BSA in PBS for 24 h at 4 °C. Secondary AB, Alexa Fluor® 488 – Goat anti-Mouse IgG AB (Invitrogen, # A11029), was applied 1 : 100 in 1% BSA in PBS for 2 h on a shaker. DAPI (4,6-diamidin-2-phenylindol) (Sigma, # D9542) nucleic acid staining was performed 1 : 1000 in PBS for 20 min at RT. Samples were protected from light during further procedure. After staining, MT were pre-embedded in 3% agarose, followed by paraffin embedding and sectioned as described above. Images were acquired with a ZEISS AXIO Imager.M1 (Zeiss, Feldbach, Switzerland).

### Scanning electron microscopy (SEM)

Histology sections of 4 μm thickness were prepared as described above, mounted on glass slides, deparaffinized with xylene (2 × 10 min) and air dried. The samples were then coated with 10 nm carbon using a sputter coater (EM ACE600, LEICA, Wetzlar, Germany) and imaged on a scanning electron microscope (Nova NanoSEM230, FEI, Hillsboro, US). Secondary electron (SE) and back-scattered electron (BSE) images were acquired simultaneously and subsequently combined to DDC-SEM micrographs by assigning the SE image to the red and the BSE image to the green channel in ImageJ (Fiji Version 2.0.0).

### Cell viability assay

The CellTiter-Glo® 3D Cell Viability assay kit was purchased from Promega (Dübendorf, Switzerland) for assessing the metabolic activity in 2D and 3D cell cultures. For 3D viability determination, MTs were transferred to black, flat bottom 96 well plates coated with gelatine as repellent surface, following the manufacturer protocol to determine the viability of one MT per well. Per condition, quadruplicates were analysed. Samples were incubated for 30 min at RT on a shaker in the dark, prior to luminescence recording using a Mithas^2^ LB943 Multimode Reader (Berthold Technologies GmbH & Co. KG, Bad Wildbad, Germany). For 2D viability determination, the CellTiter-Glo® kit for 2D cell viability assessment was purchased from Promega (Dübendorf, Switzerland) and used analogously to the aforementioned protocol. Interference of nanoparticle with the optical readout was excluded by measuring the interaction of increasing nanoparticle concentrations with the assay components in cell free conditions. No difference was observed in the presence of nanoparticles, indicating no significant interference.

Cisplatin (CISplatin Sandoz®, Sandoz Pharmaceuticals AG, Risch, Switzerland) and 5-Fluorouracil (5FU, Fluorouracil-Teva®, Teva® Pharma AG, Basel, Switzerland) were kindly provided by the Cantonal Hospital St. Gallen (KSSG). Cells and MT were treated with different concentrations of chemotherapeutics for at least 48 h and viability determined as described before.

### Live dead assay

Calcein-AM (Sigma, # 17783, Buchs, Switzerland) and Ethidium Homodimer-1 (EthD-1, Sigma, # 46043, Buchs, Switzerland) staining was used for live dead staining. A staining solution was prepared by adding 2 μl of Calcein AM (stock: 1 mM in DMSO) and 1 μl of EthD-1 (stock: 1 mM in DMSO) per 1 ml culture medium. MT were stained for 2 h under standard culture conditions. Prior to fluorescence imaging, MT were washed twice with PBS. A ZEISS LSM 800 with Airyscan (Zeiss, Feldbach, Switzerland) confocal laser scanning microscope was used for image acquisition. *Z*-stack images of 20 μm in *z*-dimension were taken and 20 individual images assembled to a maximum intensity project using the Zeiss ZEN 2.3 SP1 software.

### Irradiation experiments

#### MT sample preparation

MT were prepared as described above and transferred to low attachment plates (Gelatine coated commercial 96-well plates).

#### 2D cell sample preparation

For 2D cell irradiation experiments, cells were seeded 2 days before the irradiation experiment. Dependent on the cell line, different densities were seeded in 48-well plates to allow growth for at least 8 days without density derived cytotoxic effects. 2000 HeLa cells and for 5000 NHDF cells were seeded in 48-well plates. Exposure to AuNP was performed after cell attachment with an applied dose of 4 ng per cell for 24 h.

#### Irradiation

Cell culture medium was allowed to equilibrate to RT for about 30 min prior to the irradiation experiments. X-rays were generated using a SEIFERT X-ray Tubehousing (ISOVOLT 420/10, SEIFERT, Hamburg, Germany) at an acceleration voltage of 150 kV and 20 mA current with 50 cm sample distance and 12 mm aluminium filter, resulting in a dose of 0.6 Gy min^−1^ at the sample. A surrounding of a 4 cm thick Perspex plate served as a water phantom to simulate biological conditions. The relatively large source to sample distance was chosen to ensure homogenous irradiation of the cell culture plate. An X-ray sensor was used to monitor radiation dose as a function of time. Calibration was performed with GAFCROMIC™ EBT^3^ self-developing dosimetry films (Ashland Advanced Materials, Bridgewater, USA). Effects on viability were assessed on day 7 after irradiation by measuring metabolic activity using CellTiter-Glo® 3D Cell Viability as described before.

### Radiation enhancement ratio (RER)

RERs^23^ were calculated using the following equation for radiation dose of 4 Gy
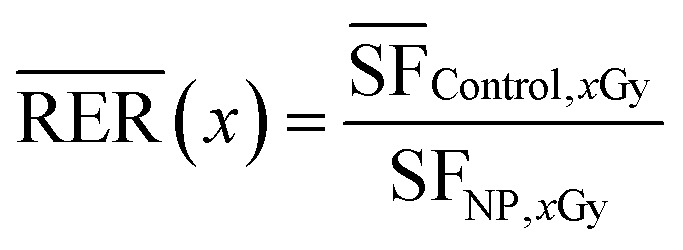
SF = survival fraction.

Different survival fractions at given irradiation dose and nanoparticle treatment, respectively.

### Inductively coupled plasma mass spectroscopy (ICP-MS)

Individual MT (≈5000 cells) or 4000 cells (if cultured in 2D) were digested in 4 ml *aqua regia* (concentrated HCl : HNO_3_ 3 : 1) in 50 ml falcon tubes for 3 h at RT. Digests were filled up to the mark with ultrapure water, resulting in a final HCl concentration of 1.8%. Depending on the exposure concentration, digests were diluted further in 1% HCl to reach Au concentrations <1 μg L^−1^ to avoid long wash-out times and to minimize matrix effects. The Au in the digests was then measured using ICP-MS (7900 ICP-MS, Agilent Technologies, Santa Clara, US). For quality control, every 20^th^ sample an external quality control multi-element standard containing 1 μg L^−1^ Au (IV100, Inorganic Ventures, Christiansburg, Virginia, US) was measured. To assure complete digestion of the AuNP, control samples spiked with a known mass concentration of 1 mg ml^−1^ AuNP dispersed in the experimental medium were digested along the experimental samples. Mass recoveries of Au in these samples were >98.5% for 50 nm and >92.5% for 5 nm AuNP. NP number concentrations were calculated from mass concentrations assuming AuNP being perfectly spherical with a density of 19.3 g cm^−3^ at RT. Mass concentrations and NP per cell are given in ESI Table S2.†

## Results and discussion

**Fig. 1:**
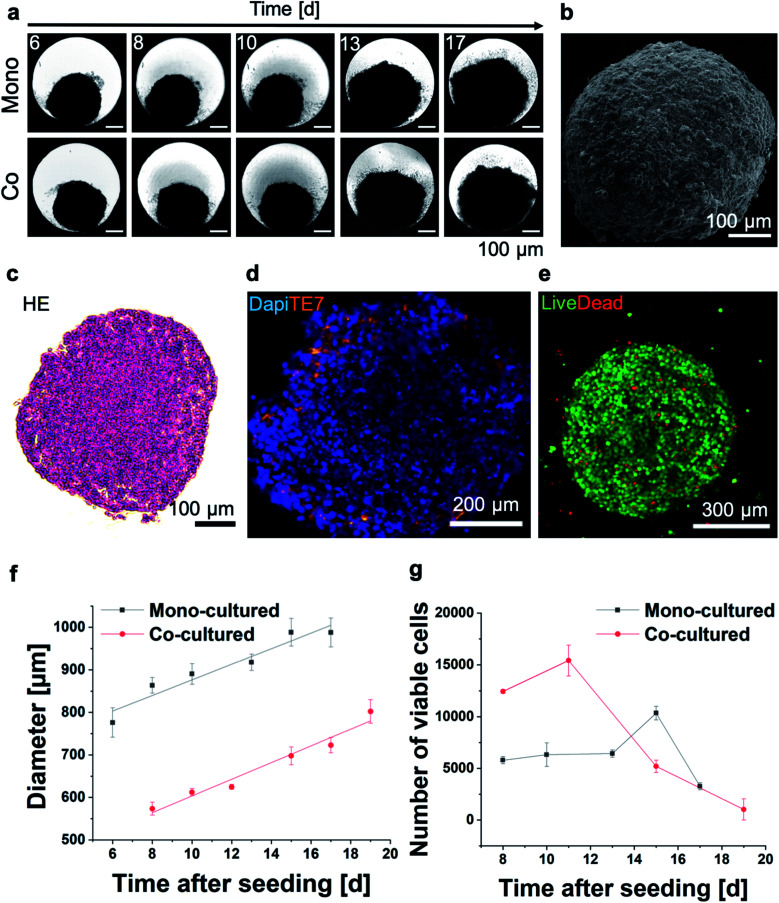
(a) Optical micrographs of mono- or co-cultured HeLa and HeLa/NHDF MT as a function of time. (b) Scanning electron micrograph of an entire mono-cultured MT. (c) Haematoxylin Eosin (HE) stained histological section of a HeLa MT. (d) Co-cultured MT stained with a fluorescent fibroblast-specific TE7 antibody (orange) and nucleus staining with DAPI (blue). (e) Representative optical micrograph of a live/dead stained mono-cultured MT on day 7 after seeding (live: green, dead: red). (f) MT diameter assessed based on optical image analysis and (g) number of viable cells as a function of time measured by an adenosine triphosphate (ATP) activity assay (*n* = 3).

First, 3D MT were assembled from HeLa cells using the well-established scaffold-free hanging-drop technique. In addition to mono-cultures, HeLa MT can optionally be co-cultured in presence of normal human dermal fibroblasts (NHDF) to mimic surrounding tissues. Following optimization of the initial cell number in order to obtain stable spheroids, HeLa or HeLa/NHDF cells were assembled into compact, stable and spherical MT and cultured for up to 17 days either as mono- or co-cultured MT, respectively (Fig. 1a and b). Hematoxylin–Eosin (HE) stained MT sections indicated a homogeneous morphology from the outside to the centre of the MT on day 7 after seeding (Fig. 1c). When NHDF were added to the culture on day 3, HeLa/NHDF MT formed with characteristics similar to the mono-cultured HeLa MT, but encapsulated by a fibroblast shell. For the HeLa/NHDF MT, the fibroblast layer surrounding the HeLa core of the co-cultured MT was visualized using the fibroblast specific antibody TE7 (Fig. 1d). The average fibroblast shell thickness was estimated based on analysis of fluorescence microscopy images as 45 ± 13 μm, corresponding to approximately 5 layers of cells. Live/dead staining indicated a low number of dead cells within the mono-cultured (Fig. 1e) and co-cultured MT on day 12. Growth curves showed a linear increase in diameter of both MT types as a function of time (Fig. 1f). Counterintuitively, the size of co-cultured MT from day 6 onwards was consistently smaller than the mono-cultured MT (Fig. 1f), despite the initially higher cell number in the cell culture (Fig. 1g) and the higher number of viable cells. It has been reported previously that culturing cells in 3D can change the size and morphology of cells due to a higher complexity, such as cell–cell interactions and tissue-like communication and density.^24^ Because of potential intratissural oxygen and nutrient penetration limitations^25^ the viability of both MT types was assessed as a function of time (Fig. 1g) to confirm their viability during prolonged culturing (up to 17 days). In contrast to the increasing tissue diameter, cell viability measurements only showed a limited increase of viable cell number as well as a reduction in viable cell numbers after day 16 for the mono-cultured and after day 12 for the co-cultured MT. The limited growth of the MT in long-term cultures is in good agreement with previous reports and shows the limitations of the MT size as an endpoint for assessing MT viability.^25^

**Fig. 2:**
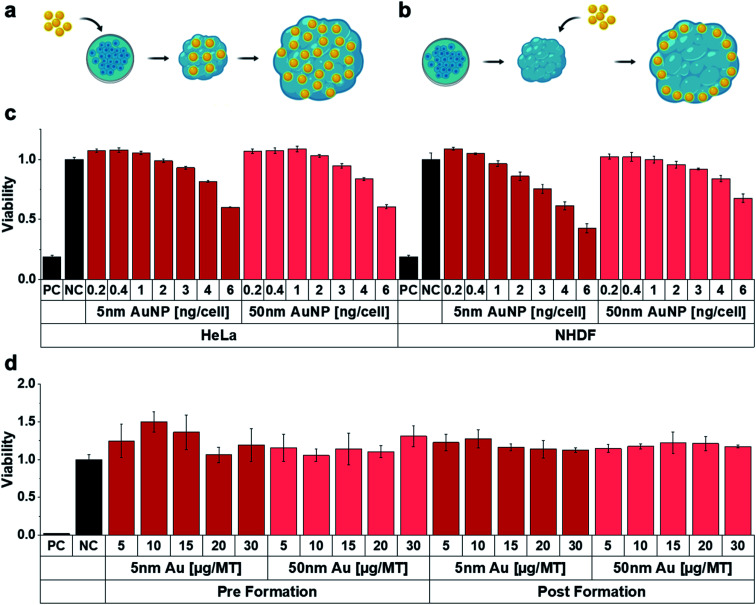
Scheme of NP exposure (a) pre- and (b) post-formation of the MT. (c) Cell viability in conventional 2D cell cultures as a function of AuNP concentration for 24 h exposure. (d) Cell viability of MT treated with AuNP for 24 h as function of NP size, applied dose and administration route. The administered dose of 1 ng per cell in 2D cell culture experiments corresponds to 5 μg per MT (representative of *n* = 3).

Following the MT assembly, the interaction of the MT with AuNP was assessed and compared to HeLa (and NHDF) cells grown in conventional 2D cell cultures (Fig. 2). Characterization data on the gold nanoparticles (AuNP, 5 and 50 nm) can be found in Table S1.† While we used widely available citrate stabilized AuNPs, surface functionalization might be introduced in order to favourably influence blood circulation times or uptake and penetration into target tissues. After excluding interferences with the luminescence readout, cell viability was assessed both in HeLa and NHDF 2D and 3D cultures as a function of NP concentration. Two administration routes were investigated to account for the well-documented poor tissue penetration of (larger) nanoparticles, which typically only accumulate in the outermost cell layer of 3D MT.^26^ The 3D MT were exposed to the NP either before or after their assembly (further termed pre-formation (Fig. 2a) and post-formation (Fig. 2b) MT, respectively). These two distinctly different exposure scenarios allowed to investigate the influence of the NP distribution within the tissue on radio-enhancement efficacy by creating a scenario where nanoparticle uptake is not limited by tissue penetration in the case of pre-formation exposure.

The 2D cultures showed a dose-dependent decrease in viability for both 5 and 50 nm AuNP in both cell lines (Fig. 2c). In contrast, viability in 3D MT cultures was not detectably compromised, regardless of whether MT were exposed pre- or post-formation (Fig. 2d). The reduced toxicity observed for the MT exposed post-formation compared to 2D may be explained by reduced nanoparticle-cell contact. However, reduced toxicity was also observed in MT exposed pre-formation, which indicates that the reduced cytotoxicity cannot solely be attributed to the reduced nanoparticle-cell contact, suggesting higher robustness of cells cultured in 3D environments, which is in line with previous work.^27^ Similarly, the cells cultured in the MT also showed higher resistance against chemotherapeutic drugs (cisplatin and 5-Fluoruracil (5-FU)) compared to 2D monolayers (Fig. S1†). While viability was severely affected by the chemotherapeutics in the 2D cultures (Fig. S1a and b†), the effects were significantly attenuated in the 3D MT (Fig. S1c†). In 3D, only the highest doses of cisplatin and 5FU reduced the viability after 96 h of treatment. The increased viability in MT relative to untreated control upon both chemotherapeutics at lower concentrations and exposure times might be explained by the well-known hormesis effect^28^ where stress induces proliferation as survival strategy of the cellular system. The reduced susceptibility of the cells in 3D compared to 2D illustrates the dependence of the cellular response on the cell microenvironment. Our results are well in line with several other studies^29–31^ that attribute differences in response between 3D and 2D to the altered, more tissue-like cell phenotypes observed in 3D.^32^

**Fig. 3:**
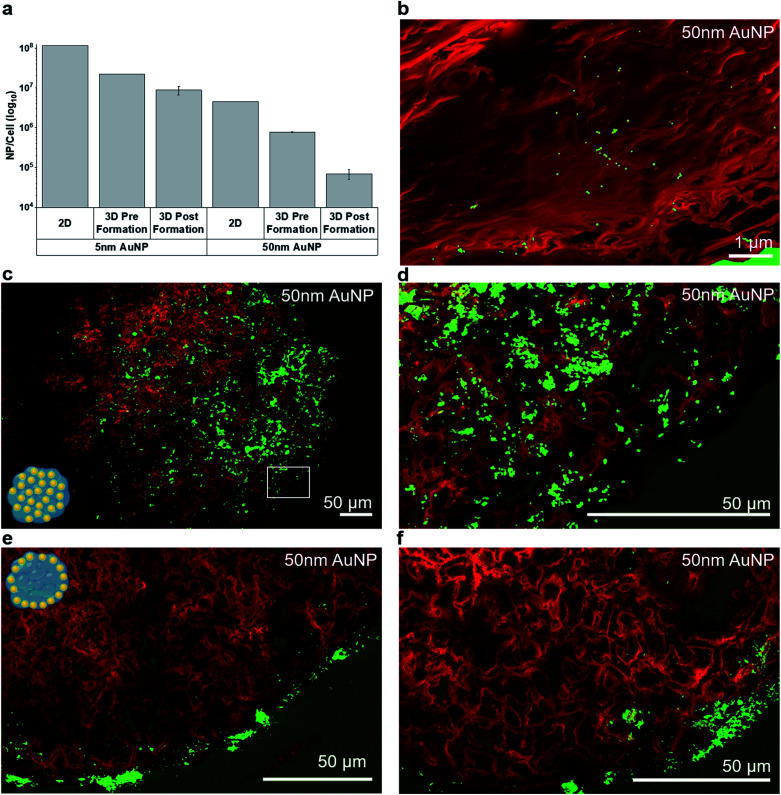
(a) NP uptake into 3D MT measured by elemental analysis using ICP-MS (representative of *n* = 2). (b–f) Intratissural AuNP distribution in mono-cultured MT assessed by density dependent color scanning electron microscopy (DDC-SEM). Red color was assigned to the secondary electron (SE) image and green to the back scattered electron (BSE) image. (b) At high magnifications, individual AuNP can be detected in histological tissue sections based on the BSE signal. (c–f) Overview and detail images illustrating the two distinctly different intratissural NP distributions, *i.e.* (c and d) pre-formation and (e and f) post-formation in mono-cultured MT.

For subsequent experiments, the highest sub-toxic AuNP dose (20 μg per MT, corresponding to 4 ng per cell) was selected. The NP uptake into MT was quantified by inductively coupled plasma mass spectroscopy (ICP-MS) (Fig. 3a). As expected, the uptake of the AuNPs into MT exposed post-formation was limited compared to MT exposed pre-formation. Uptake of the AuNP into the tumor spheroids was in good agreement with a recent study, which compared the uptake of AuNP into cell monolayers, tumor spheroids and tumor tissue *in vivo*.^33^ We found that, depending on NP size and exposure setting, between 10^4^ and 10^7^ AuNP were taken up on average per cell in the tumor MT, for 50 nm and 5 nm AuNP respectively. This finding is well in line with the range reported by Huang *et al.*^33^ for AuNP (2–15 nm), where 30 × 10^9^ AuNP were taken up per MT (one MT corresponding to ≈600 cells) translating into approximately ≈10^7^ AuNP per cell. Studies have also reported that the cellular uptake is highly dependent on the NP size due to size-dependent uptake mechanisms.^34^ Uptake dynamics and subcellular localization of the AuNP maybe strongly dependent on surface properties (including functionalization) and properties of the resulting protein corona.^35^ While we observed a higher mass uptake for 50 nm AuNP compared to 5 nm AuNP in both 2D as well as in 3D, particle number concentrations were 20 to 30-fold higher for 5 nm compared to 50 nm AuNP (Table S2†), which is well in line with recently published work.^36^ Note that fewer particles are found in the 3D pre-formation administration compared to the 2D scenario. This can be attributed to the longer cell cultivation time and possibly to nanoparticle exocytosis.^37^ The relative uptake of the 5 nm and 50 nm AuNP per cell was comparable between 2D and 3D, if NP were added pre-formation to tissue (28-fold *vs.* 22-fold higher uptake for 5 nm compared to 50 nm respectively), however, overall uptake was ≈5-fold (pre-formation) to ≈50-fold (post-formation) lower in 3D compared to 2D (for both AuNP sizes).

In addition to total NP uptake, we also assessed NP distribution within the MT by DDC-SEM based on backscattered electron (BSE) and secondary electron (SE) micrographs (Fig. 3b–f). While scanning electron microscopy only allows a semi-quantitative analysis of the NP distribution, the spatial resolution is superior to commonly used techniques, such as laser ablation inductively coupled plasma mass spectroscopy (lateral resolution ∼ 1 μm).^26^

Single 50 nm AuNP were readily identified in histological sections (Fig. 3b). As expected, MT exposed pre-formation showed a rather uniform NP distribution within the MT (Fig. 3c, d and S3a†). The occasionally observed non-symmetric nanoparticle distribution within the pre-formation MT (*e.g.*Fig. 3c and d) may be attributed to non-uniform tissue growth or occasional sectioning artefacts where large AuNP agglomerates are smeared across the tissue section. A similar distribution pattern was observed in the HeLa core of co-cultured MT exposed pre-formation and only very few NPs were found in the fibroblast layer (Fig. S3c†). In contrast, MT exposed post-formation showed limited AuNP penetration and significantly lower uptake (Fig. 3e and f), in agreement with the ICP-MS results. The majority of post-formation exposed 5 nm AuNP was located within the outer 20 μm thick peripheral layer of the MT, whereas the 50 nm-sized AuNP mainly adhered to the surface of the MT (Fig. 3e and f). While DDC-SEM allows large-area imaging at high resolution, the use of the backscattered electron signal does not allow distinction between extracellular and intracellular nanoparticles. To confirm intracellular uptake of AuNP, scanning transmission electron microscopy (STEM) of 100 nm thin sections was performed and enabled visualization of accumulated AuNP in membrane- and non-membrane-bound intracellular vesicles (Fig. S2†). However, the field-of-view for conventional transmission electron microscopy methods unfortunately is limited. Note that intratissural distribution as well as intracellular nanoparticle agglomeration and clustering are a highly dynamic process^38^ which may, in addition to cell phenotype, nanoparticle dose and intracellular localization, influence radio-enhancement performance.

**Fig. 4:**
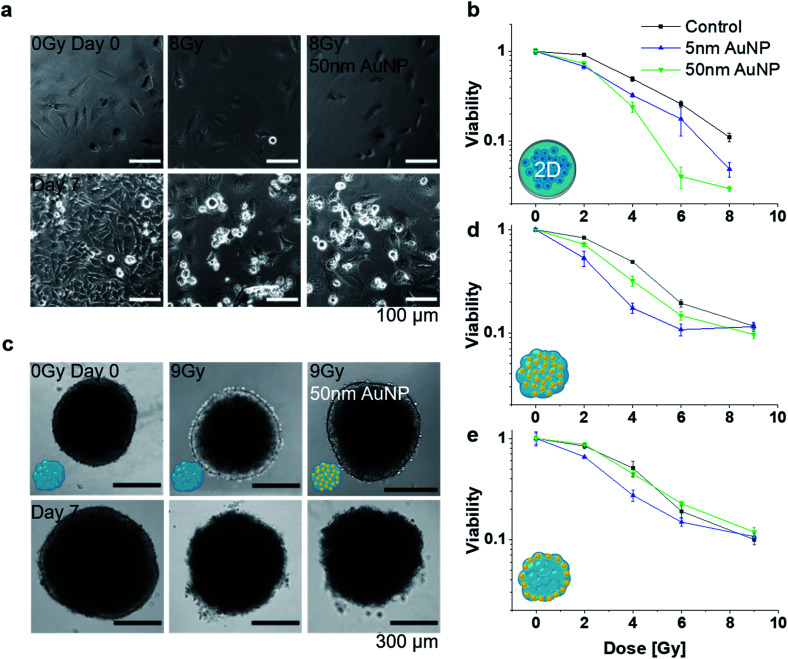
(a) Optical micrographs of 2D cell cultures exposed to an X-ray dose of 0 (control) and 8 Gy in presence or absence of 50 nm AuNP on day 0 and 7 after irradiation and (b) corresponding survival fractions on day 7 after irradiation for 5 and 50 nm AuNP treatment. (c) Optical micrographs of 3D mono-cultured MT exposed to an X-ray dose of 0 (control) and 9 Gy in presence or absence of 50 nm AuNP administered pre-formation. Corresponding survival fraction for cells in 3D mono-cultured MT with AuNP exposure (d) pre- or (e) post-formation. (*n* = 3 for all survival fraction analysis).

Following the characterization of the AuNP distributions within the MT, radio-enhancement effects were investigated for the different particle sizes and tissue distributions, and compared to 2D cell cultures. Radio-enhancement effects were first investigated in 2D cell cultures using HeLa (Fig. 4a and b) and NHDF cells (Fig. S4†). Radiation enhancement ratios (RER)^23^ were used to describe the relation of the viability of cells exposed to NP compared to non-exposed cells at a certain irradiation dose. At 4 Gy irradiation, RER between 1.52 ± 0.06 and 2.10 ± 0.33 (Table 1) were found for 2D HeLa cells treated with 5 nm and 50 nm AuNP, respectively. These RER results are in good agreement with values reported in the literature (*e.g.*, Butterworth *et al.*^39^ reporting RER_2Gy_ between 0.8 and 2 depending on the cell line for 1.9 nm AuNP, and Khoshgard *et al.*^40^ reporting RER_2Gy_ of 1.65 for HeLa cells containing 52 nm AuNP). After confirming the radio-enhancement properties of the selected NP in conventional 2D cultures, studies were performed in 3D mono-cultured MT. First, the radio-enhancement effect in MT exposed pre-formation was investigated. For both AuNP sizes, an enhancement effect was observed with a RER_4Gy_ of 2.85 ± 0.29 for 5 nm AuNP and 1.55 ± 0.16 for 50 nm AuNP in MT exposed pre-formation (Table 1 and Fig. 4c and d). In MT exposed post-formation, only the treatment with 5 nm AuNP lead to a significant radio-enhancement effect with a RER_4Gy_ of 1.87 ± 0.12 (Table 1). Microtissues treated with 50 nm AuNP post-formation showed a survival behaviour indistinguishable from the nanoparticle-free controls (Fig. 4e), with a RER_4Gy_ of 1.15 ± 0.13, which can likely be attributed to the poor penetration of the 50 nm AuNP into the MT, as indicated in Fig. 3e and f. Comparing the radio-enhancement effects in 2D and 3D cultures, it stands out that the overall survival in 2D is lower compared to 3D for the same radiation dose, supporting the hypothesized increased robustness in tissue-like structures. While the radio-enhancement effects for 5 nm AuNP were comparable between 2D and 3D, a distinctly different response was observed for the 50 nm AuNP. The 50 nm AuNP were most efficient in 2D, however, no significant dose-enhancement was observed in the MT model, which highlights the importance of models with tissue-like features early in the development of radio-enhancers. While radio-enhancement effects can be quantitatively assessed in the mono-cultured MT and contextualized with AuNP uptake and distribution, the effect on the co-culture tissues cannot be reliably assessed due to the inability to quantitatively distinguish the effects on the NHDF and the HeLa cells. However, the radio-enhancement effects appear to be attenuated in the NHDF mono-cultures (see Fig. S4†) compared to the HeLa cells, indicating that the cancerous tissues may indeed be more affected than the healthy fibroblasts. Note that the keV energies (instead of clinically used MeV) and the relatively low dose rates (approximately 0.6 Gy min^−1^ compared to the clinically used 4–6 Gy min^−1^) in the present irradiation setting may limit the direct translatability of the findings to the clinical setting. While the linear quadratic characteristics of our survival curves indicate significant radiation damage, dose rates lower than ours may lead to partial DNA repair already during irradiation and hence to an underestimation of the radiation effects.

**Table 1:** Radiation enhancement ratios (RER) for 4 Gy radiation for 2D and 3D HeLa cell culture with AuNP exposure pre- or post-formation of the MT

	RER_4Gy_
2D 5 nm AuNP	1.52 ± 0.06*
2D 50 nm AuNP	2.10 ± 0.33*
3D 5 nm AuNP, pre-formation	2.85 ± 0.29*
3D 50 nm AuNP, pre-formation	1.55 ± 0.16*
3D 5 nm AuNP, post-formation	1.87 ± 0.12*
3D 50 nm AuNP, post-formation	1.15 ± 0.13*

## Conclusions

Taken together, we present a tumor spheroid model along with methods to assess NP uptake and distribution with nanometric resolution in a label-free approach, which in turn allows putting radio-enhancement findings into context. The MT model allows studying NP penetration, diffusion, retention and radio-enhancement in *in vitro* systems that more closely simulate *in vivo* architectures. Cells are less susceptible to drugs and NP treatments in the 3D MT environment compared to 2D tissue cultures, which is in line with the oftentimes reduced effects observed *in vivo* compared to *in vitro* cell cultures. Additionally, and in contrast to the 2D cell culture model, the MT model adequately reproduces the reduced tissue penetration of larger NP observed *in vivo*,^33^ which leads to attenuated therapeutic efficacy. The present *in vitro* model allows a high-throughput optimization of nanoparticle-enhanced radiotherapy conditions (optionally also in combinations with chemotherapeutics) under well-standardized conditions supported by high-resolution analytical imaging. However, subsequent *in vivo* studies are imperative to study more complex phenomena in presence of a functional immune system. Nonetheless, because of its tissue-mimicking properties, high robustness and simplicity, the approach deserves consideration in the oftentimes challenging preclinical translation from 2D cell culture to *in vivo*.

## Conflicts of interest

There are no conflicts to declare.

## Supplementary Material

NA-002-D0NA00256A-s001
