# Supplementary material for: Uptake, distribution and radio-enhancement effects of gold nanoparticles in tumor microtissues
Source: Nanoscale Adv. 2020 Jun 2;2(7):2992–3001. doi: 10.1039/d0na00256a (PMC9417636; doi:10.1039/d0na00256a)
Supplement: NA-002-D0NA00256A-s001 [file NA-002-D0NA00256A-s001.pdf]

Electronic Supplementary Material (ESI) for Nanoscale Advances.
This journal is © The Royal Society of Chemistry 2020

Supporting Information

Uptake, Distribution and Radio-enhancement Effects of Gold Nanoparticles in Tumor Microtissues

Anna L. Neuer,[a,b] Lukas R.H. Gerken,[a,b] Kerda Keevend,[a,b] Alexander Gogos,[a] Inge K. Herrmann *[a,b]

[a.] Laboratory for Particles Biology Interactions, Swiss Federal Laboratories for Materials Science and Technology (Empa), Lerchenfeldstrasse 5, CH-9014, St. Gallen, Switzerland.
[b.] Nanoparticle Systems Engineering Laboratory, Institute of Process Engineering, Department of Mechanical and Process Engineering, ETH Zurich, Sonneggstrasse 3, CH-8092 Zurich, Switzerland.

*inge.herrmann@empa.ch; ingeh@ethz.ch; +41 (0)58 765 7153

Table S1: Physico-chemical characterization of the AuNP purchased from nanoComposix (values given by manufacturer excl. zeta potential measured according to a protocol by EU-NCL with 10% PBS).

	**5 nm AuNP**	**50 nm AuNP**
**Diameter**	5±0.7 nm	52±0.7 nm
**Hydrodynamic diameter**	NA	62 nm
**Zeta potential**	-43.2±0.8	-41.6±0.9
**Particle surface**	Sodium citrate	Sodium citrate
**Dispersant**	H_2O	H_2O

Table S2: AuNP uptake assessed by ICP-MS in 2D and 3D for mono- and co-cultured MT as well as both AuNP administration routes. Values represent the mean of 3 replicates. Number concentrations were calculated from the determined mass concentrations assuming a gold density of 19.3 g cm^{-3} (representative of n=2).

**2D**		
**5 nm AuNP**	0.144 ng/cell<br>$1.15 \cdot 10^{8}$ NP/cell	
**50 nm AuNP**	5.77 ng/cell<br>$4.58 \cdot 10^{6}$ NP/cell	
**3D Mono-Cultured**	**Pre Formation**	**Post Formation**
**5 nm AuNP**	0.028±0.0002 ng/cell<br>$(22.33\pm0.2) \cdot 10^{6}$ NP/cell	0.011±0.003 ng/cell<br>$8.83 \cdot 10^{6}$ NP/cell
**50 nm AuNP**	0.984±0.020 ng/cell<br>$(0.78\pm0.02) \cdot 10^{6}$ NP/cell	0.087±0.027 ng/cell<br>$0.07 \cdot 10^{6}$ NP/cell
**3D Co-Cultured**	**Pre Formation**	**Post Formation**
**5 nm AuNP**	0.034±0.004 ng/cell<br>$(27.11\pm3.29) \cdot 10^{6}$ NP/cell	0.009±0.0001 ng/cell<br>$7.13 \cdot 10^{6}$ NP/cell
**50 nm AuNP**	1.526±0.007 ng/cell<br>$(1.21\pm0.59) \cdot 10^{6}$ NP/cell	0.028±0.016 ng/cell<br>$0.02 \cdot 10^{6}$ NP/cell

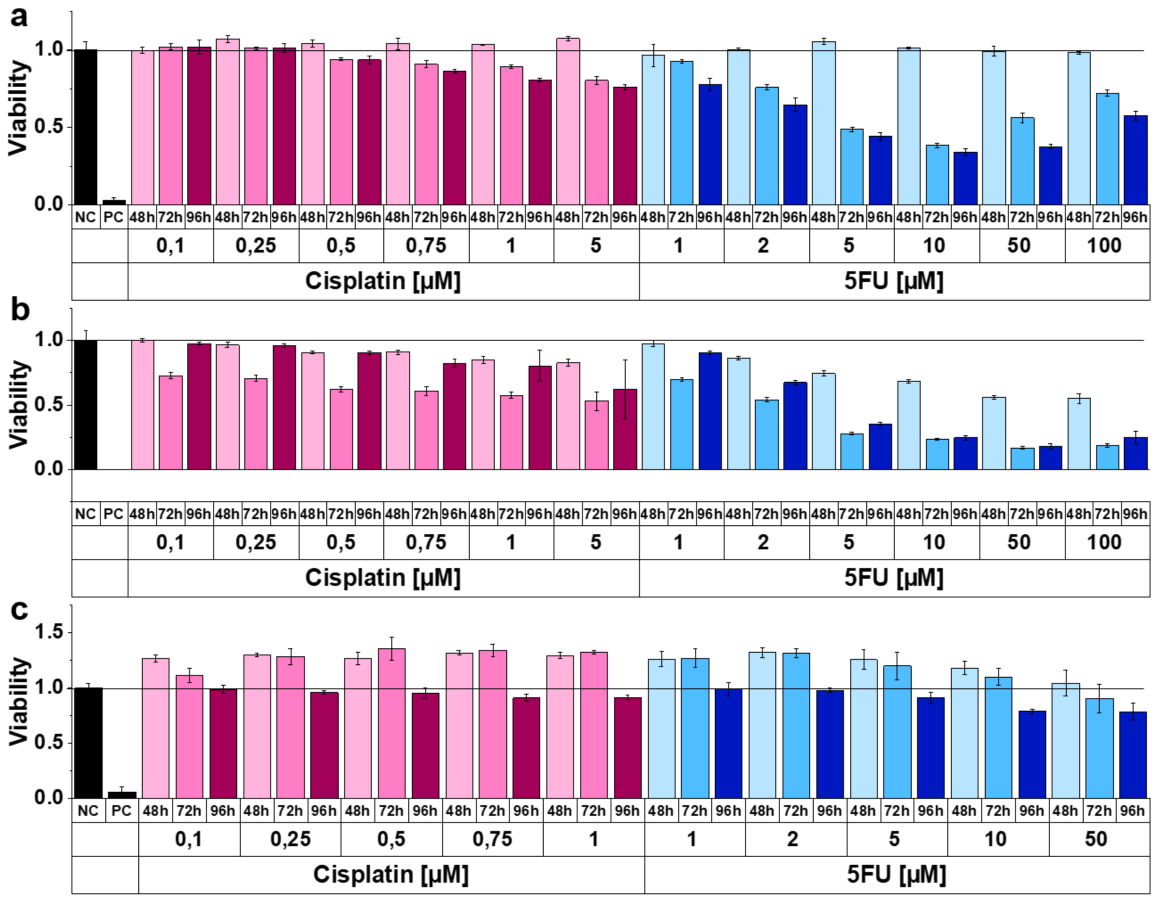

Figure S1: Cell viability of HeLa and NHDF cells in 2D and in 3D mono-cultured MT exposed to chemotherapeutic drugs cisplatin and 5FU as a function of applied dose and time. (a) 2D HeLa cells, (b) 2D NHDF cells and (c) 3D mono-cultured HeLa MT. All experiments n=3

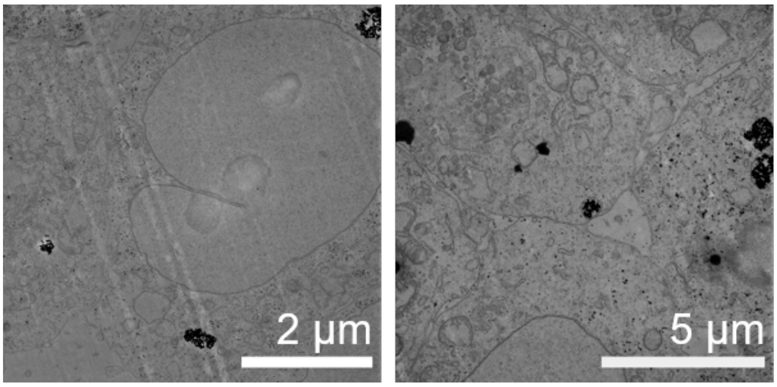

Figures S2: Scanning transmission electron micrographs of 100 nm thin sections of mono-cultured Hela MT containing 50 nm AuNP show agglomerates enclosed in membrane and non-membrane-bound vesicles.

Scanning transmission electron microscopy (STEM).

For STEM, 8-10 MT per condition were pooled after fixation and stained with 2% osmium tetroxide for 1 hour at RT. Then, they were gradually dehydrated with increasing EtOH concentrations followed by epoxy resin (EPON 812, Sigma Aldrich) embedding according the manufacturer protocol. Resin blocks were cured at 60°C for 72h. Ultrathin sections of 100 nm thickness were prepared using an ultramicrotome Leica EM UC6 (Leica Mikrosysteme GmbH, Austria). STEM imaging was performed with a FEI Helios 660 G3 UC Focused Ion Beam (FIB)/SEM with STEM function.

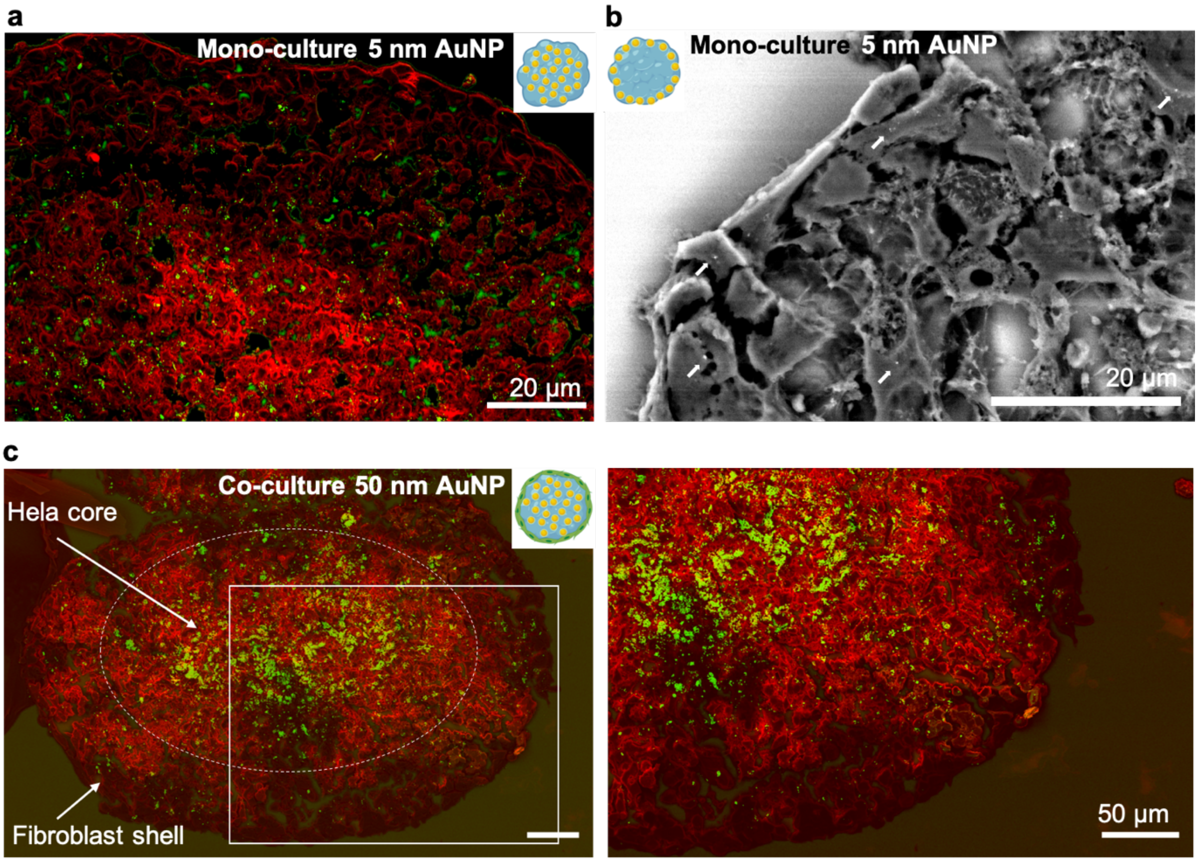

Figure S3: Intratissural distribution of AuNP. (a) DDC-SEM overview image showing the intratissural distribution of 5 nm AuNP in a histological section of mono-cultured HeLa MT (scale bar 20 µm). (b) BSE image of mono-cultured MT with 5 nm AuNP post-formation exposure with arrows pointing to NP. (c) DDC-SEM micrographs of co-cultured MT with HeLa core and NHDF shell were 50 nm AuNP were added to the HeLa cells prior to the MT formation (pre-formation, scale bars 50 µm).

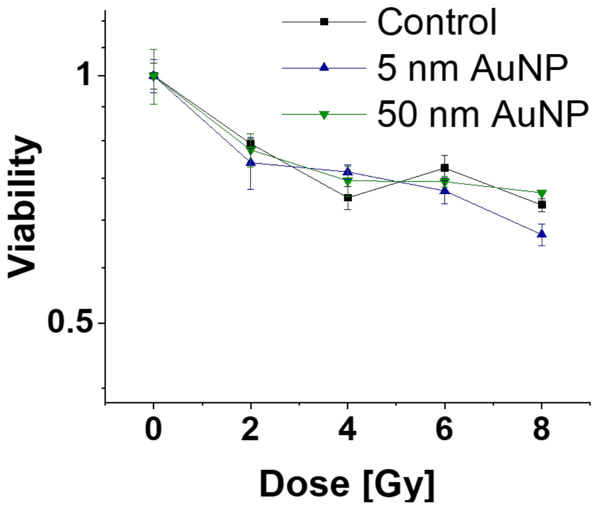

Figure S4: Survival fraction of 2D NHDF cell culture exposed to X-ray irradiation in presence or absence of AuNP (n=3).
